# Report of the Prizes of the Academy of Medicine of Paris for 1860

**Published:** 1861-03

**Authors:** 


REPORT OF THE PRIZES OF THE ACADEMY OF MEDICINE OF PARIS FOR 1860.—The Academy Prize of 1000 francs, on "the Means of Avoiding the Accidents which may be Produced by the Employment of Ether and Chloroform, and the Best Methods of Remedying these Accidents," has not been awarded; but Dr. Faure is encouraged by the sum of 600 francs. The Portal Prize, on "Vascular Obstructions of the Circulatory System of the Lung," was not awarded; but to the authors of the only work which was presented, Drs. Charcot and Benjamin Bell, was given 600 francs, the value of the prize. For the Civrieux Prize of 2400 francs, on "the Influence of Chloro-anæmia on Nervous Excitement," there were sixteen competitors; but their memoirs were not adjudged worthy of the prize. Dr. Simon was, however, recompensed with 900 francs, Dr. Mordet with 900 francs, and Dr. Zurkowski with 600 francs, and four other competitors were honorably mentioned. The Barbier Prize was not awarded, none of the memoirs having fulfilled the required conditions. The Lefèvre Prize of 1500 francs, on "the Diagnosis and Treatment of Melancholy," was awarded to Dr. Semelaigne. The Capuron Prize of 1000 francs, on "Puerperal Paralysis," was carried off by Dr. Imbert-Gourbeyre. The Orfila Prize, on "Poisonous Mushrooms," was not awarded.

